# Supporting at-risk older adults transitioning from hospital to home: who benefits from an evidence-based patient-centered discharge planning intervention? Post-hoc analysis from a randomized trial

**DOI:** 10.1186/s12877-020-1494-3

**Published:** 2020-03-02

**Authors:** Véronique Provencher, Lindy Clemson, Kylie Wales, Ian D. Cameron, Laura N. Gitlin, Ariane Grenier, Natasha A. Lannin

**Affiliations:** 1 School of Rehabilitation, Faculty of Medicine and Health Sciences, Université de Sherbrooke Research Centre on Aging, 3001 12e Avenue Nord, Sherbrooke, Québec, J1H 5N4 Canada; 2 Faculty of Medicine & Health, The University of Sydney, Sydney, 2006 Australia; 3 School of Health Sciences, University of Newcastle, Callaghan, 2308 Australia; 4 John Walsh Centre for Rehabilitation Research, Faculty of Medicine and Health, University of Sydney, Sydney, Australia; 5 College of Nursing and Health Professions, Drexel University, 1601 Cherry Street, Philadelphia, PA 19102 USA; 6 Research Center on Aging, 1036 Belvédère Sud, Sherbrooke, Québec, Canada; 7 Department of Neuroscience, Central Clinical School, Monash University, 99 Commercial Road, Melbourne, 3004 Australia; 8 Alfred Health, 55 Commercial Road, Melbourne, 3004 Australia

**Keywords:** Discharge planning, Home visit, Occupational therapy, Older adults, Cognitive impairment, Rehabilitation

## Abstract

**Background:**

Subgroups of older patients experience difficulty performing activities of daily living (ADL) following hospital discharge, as well as unplanned hospital readmissions and emergency department (ED) presentations. We examine whether these subgroups of “at-risk” older patients benefit more than their counterparts from an evidence-based discharge planning intervention, on the following outcomes: (1) independence in ADL, (2) participation in life roles, (3) unplanned re-hospitalizations, and (4) ED presentations.

**Trial design and methods:**

This study used data from a randomized control trial involving 400 hospitalized older patients with acute and medical conditions, recruited through 5 sites in Australia. Participants receive either HOME, a patient-centered discharge planning intervention led by an occupational therapist; or a structured in-hospital consultation. HOME uses a collaborative approach for goal setting and includes pre and post-discharge home visits as well as telephone follow-up. Characteristics associated with higher risks of adverse outcomes were recorded and at-risk subgroups were created (mild cognitive impairment, walking difficulty, comorbidity, living alone and no support from family). Independence in ADL and participation in life roles were assessed with validated questionnaires. The number of unplanned re-hospitalizations and ED presentations were extracted from medical files. Linear regression models were conducted to detect variation in response to the intervention at 3-months, according to patients’ characteristics.

**Results:**

Analyses revealed significant interaction effects for intervention by cognitive status for unplanned re-hospitalization (*p* = 0.003) and ED presentations (*p* = 0.021) at 3 months. Within the at-risk subgroup of mild cognitively impaired, the HOME intervention significantly reduced unplanned hospitalizations (*p* = 0.027), but the effect did not reach significance in ED visits. While the effect of HOME differed according to support received from family for participation in life roles (*p* = 0.019), the participation observed in HOME patients with no support was not significantly improved.

**Conclusions:**

Findings show that hospitalized older adults with mild cognitive impairment benefit from the HOME intervention, which involves preparation and post-discharge support in the environment, to reduce unplanned re-hospitalizations. Improved discharge outcomes in this at-risk subgroup following an occupational therapist-led intervention may enable best care delivery as patients transition from hospital to home.

**Trial registration:**

The trial was registered before commencement (ACTRN12611000615987).

## Background

Most hospitalized older adults wish to return home and reengage in meaningful activities [1]. However, hospital-associated deconditioning often results in increased difficulty performing activities of daily living (ADL) [2] and resuming valued life-roles in the months following discharge [3]. Those who report unmet needs related to new difficulties in ADL after they return home are at higher risk of hospital and emergency department (ED) admissions [4, 5]. Furthermore, past studies suggested that some physical, psychological and social characteristics increased the risk of these adverse discharge outcomes in older patients [6, 7]. In particular, difficulty to walk, comorbidities, cognitive impairment, living alone and lack of social support have been identified as strong predictors of readmissions within 30 days post-discharge [8–14]. While not all readmissions are avoidable, a large proportion (27–76%) is thought to be preventable if best-practice discharge preparation and practices are followed [15]. Targeting these at-risk patients to improve their daily functioning after returning home by providing them with optimal discharge planning may, in turn, reduce unplanned readmissions.

Optimal hospital discharge planning should provide older adults and their care partners, if relevant, with appropriate support (equipment, home modifications, and services) which can promote independent functioning and reduce safety risks after discharge [16]. Previous research suggests that home visits, through the assessment of the patients’ abilities in their own environment [17] may contribute to post-discharge independence in ADL [18] and decrease readmissions in older adults [19, 20], especially when combined with phone follow-up [21, 22]. To be effective, interventions must provide care tailored to older adults’ specific needs during the transition from hospital to home and actively support patients and family engagement in the discharge process [23, 24]. However, in many countries, hospital and community care practices tend to be fragmented [25] and goal setting is rarely established in partnership with patients’ and families’ during discharge planning, in turn increasing the risks of stress and frustration as well as inappropriate care management when returning home [26, 27].

To fill this gap, the HOME discharge planning intervention was developed to merge best practices into an innovative discharge planning occupational therapy intervention which included goal-setting, equipment prescription, home visits and telephone follow-up [28]. Occupational therapy-based interventions have the potential to reduce readmissions by addressing patients’ context-specific functional and social needs [29]. By understanding patients’ experience and situations, HOME facilitates the development of a collaborative process, which considers the match between patient and environment, helps patients take ownership of their discharge-related goals as well as the means to achieve them. The HOME intervention was assessed in a randomized control trial (RCT) involving 400 older patients with varied medical conditions treated in five acute Australian hospitals [28]. Consistent with other evidence-based interventions [30, 31], no between-group differences were found between HOME compared to the in-hospital occupational therapy consultation on the main outcomes (independence in ADL, participation in life roles, and hospital and ED readmissions) in this trial [28].

One hypothesis is that the HOME intervention may improve discharge outcomes for specific subgroups of older patients who may be at most risk for negative health outcomes. This hypothesis is supported by an RCT conducted by Ritchie et al. (2016) [32], which found that an intervention aimed to support care transition from hospital to home (e-coach technology) was beneficial for some patients but not others, with those with chronic obstructive pulmonary disease benefiting, but not those with cardiac problems. To our knowledge, little is known about who benefits most from hospital discharge planning interventions and how responsive are those at higher risk of poor outcomes upon returning home to these [33]. Identification of subgroups for whom this patient-oriented intervention works and on which discharge outcomes will thus shed light on the results obtained from the overall sample in the Clemson et al. study [28] and support best care delivery decision-making.

Therefore, this post-hoc analysis of the Clemson et al. study [28] aimed to examine whether subgroups of older patients with characteristics associated with higher risks of discharge adverse outcomes (including difficulty walking, higher comorbidities, mild cognitive impairment, living alone, and no support from family) benefit from the HOME intervention on independence in activities of daily living (ADL), participation in life roles, as well as unplanned readmissions to hospital and ED presentations.

## Methods

### Study sample and procedures

All study procedures are reported elsewhere (see Wales et al., 2002 [34] for protocol, and Clemson et al.*,* 2016 [28] for full study details). Briefly, participants were recruited from medical and acute care wards in five hospitals between June 2011 to March 2014 in Melbourne and Sydney, Australia. Study participants were 70 years and older, without significant cognitive impairment (less than 5 errors on the Short Portable Mental Status Questionnaire (SPMSQ)) [35] and English speaking. They were excluded if they showed major mobility problems (score < 5/7 on locomotion subscale of the Functional Independence Measure score (FIM)) [36], had significant comorbidities (as predicted by age-adjusted cut-offs for Charlson Comorbidity Index (CCI) score [37]), had received a home visit by an occupational therapist in the 6 months prior to admission, or had been referred to rehabilitation at discharge.

Participants were individually randomized to receive either the HOME intervention or a structured in-hospital consultation, stratified by site and age. The HOME intervention includes four steps: 1) establishment of a hospital-based partnership with patient and family for goal setting and problem solving; 2) pre-discharge home assessment to address safety issues and home modifications with patient and family; 3) post-discharge home assessment and in-home training to address unmet needs; and 4) follow-up telephone calls to provide ongoing support to increase independence for participants and families and ensure required services have been accessed. The in-hospital consultation involved a standardized assessment and consultation by an occupational therapist for planning and supporting discharge to home, inclusive of equipment prescription where clinically warranted; no occupational therapy post-discharge support was provided to the group who received the in-hospital consultation.

#### Measures

Physical, psychological and social characteristics associated with higher risks of adverse discharge outcomes were selected based on past studies [6–14]:

- *Ability to walk* was determined by the Functional Independence Measure (FIM) [36]. The FIM contains 18 items, including *walking* [38]. Ratings for *walking* item ranged from 1 (total assistance) to 7 (complete independence). Scores of 5 and 6 respectively mean that patient walks *with* or *without* supervision with a device. In the present study, the sample was split to create two subgroups: the **at-risk subgroup** refers to participants showing **difficulty to walk** (**score 5 or 6**), while the other subgroup includes those having no difficulty to walk (score 7).
- *Comorbidity* was captured by the aged-adjusted CCI [37]. This tool rates 17 comorbidities. A weighted-score (range: 1 to 6) is assigned to each category, a higher score indicating an increased severity of the condition. An age-adjusted score of CCI > 5 has been found to predict in-hospital complications [39]. In the present study, the sample was split to create two subgroups: the **at-risk subgroup** refers to participants showing a **higher comorbidity score (6 to 9)**, while the other subgroup includes those having a lower comorbidity score (5 or lower).
- *Cognitive status* was determined by the score of SSPMSQ [35]. The 10-items give a picture of the patient’s capacity for memory, orientation and calculation. The score interpretation is based on the number of errors: *0–2 errors*: normal mental functioning; *3–4 errors*: mild cognitive impairment;. Using the cut-off point of three errors, the SSPMSQ proved to be a sensitive (86.2%) and specific (99.0%) screening cognitive test among medical inpatients [40]. In the present study, the sample was split to create two subgroups: the **at-risk subgroup** refers to participants showing **mild cognitive impairment** (**3 or 4 errors**), while the other subgroup includes those having no cognitive impairment (0 to 2 errors).
- *Living alone* was self-reported (yes/no) by answering to the following question: *“Do you live alone?”* In the present study, the sample was split to create two subgroups: the **at-risk subgroup** refers to participants **living alone**, while the other subgroup includes those not living alone.
- *Support from family* was self-reported (yes/no) by answering to the following question: “*Do you receive support from family”*. In the present study, the sample was split to create two subgroups: the **at-risk subgroup** refers to participants **with no support from family**, while the other subgroup includes those having support from family.

Primary outcomes measures were *independence in ADLs* and *participation in life roles,* as well as *number of unplanned re-hospitalizations and ED visits.*

Two self-report questionnaires were used to evaluate the *independence in ADLs* and the *participation in life roles*:

- The *Nottingham Extended Activities of Daily Living (NEADL scale)* comprises 22 questions to measure the independence in the following areas of daily living: mobility, kitchen, domestic and leisure activities [41]. Each question was scored using a dichotomized scale (0: unable, 1: able). The validity of the NEADL scale has been established with hospitalized patients (stroke, hip fracture) and its reliability is good [42];
- The *Late Life Disability Index* (LLDI) includes 16 questions which assess *frequency* and *limitation* in life roles. Five-point scales were used to measure frequency (1: never, 5: very often) and limitation (1: completely, 5: not at all). Test-retest reliability of the LLDI is moderate to high [43]. Current data [44] supported the construct validity and sensitivity to change of this tool among various clinical populations of community dwelling older adults.

*Characteristics associated with higher risks of adverse discharge outcomes* were collected through file audit and interview before discharge. *Independence in ADLs* and *participation in life roles* (NEADL and LLDI) were administrated at baseline in the hospital and 3 months after discharge in the participant’ home by trained research assistant masked to group assignment. *Number of unplanned re-hospitalizations and ED visits* 3 months after discharge was extracted from medical files. Ethic approval was obtained from the hospital and university committees. The trial was registered prior the beginning of the study.

### Statistical analysis

Descriptive statistics were performed to describe the participants’ baseline characteristics, as well as outcomes measures and attrition rates. Parametric (independent t-tests) and distribution free statistics (Pearson’s chi-square, Wilcoxon) were used to compare participants’ baseline characteristics by groups (in-hospital vs HOME) [45].

Linear regression models were conducted for each of the four main outcomes (NEADL, LLDI, hospital readmissions and ED presentations) in order to detect variation in response to the HOME intervention at 3 months, according to the following patients’ characteristics groups: ability walking (with/without difficulty), comorbidities (higher/lower), cognitive status (mildly impaired/ unimpaired), living alone (yes/no), and support from family (yes/no). To test for interactions, we explore if the patients with characteristics associated with higher risks of adverse outcomes – the at-risk subgroup - responded differently to the HOME intervention than their counterparts. More specifically, one characteristic group and an interaction term between interventions (in-hospital vs HOME) were introduced for each outcome. When the interaction was found significant within a characteristic group, we explored if outcomes significantly improved following the HOME intervention in the at-risk participants.

All analyses followed an intention-to-treat model. Skewed distribution of residuals was checked. SPSS version 14.0 was used and significance level was set at *p* < .05.

## Results

### Study sample characteristics

Characteristics of the overall sample were reported elsewhere (see Clemson et al. [28] for details). Briefly, the participants (*n* = 400) were 80.5 years on average and primarily female (61.8%). They completed 12 or 13 years of education (31.5%), took five or more medications (60.8%) and were hospitalized 1 to 2 times in the last year. There was no significant difference between groups on these sociodemographic characteristics. Proportion of “at-risk” participants in the HOME (*n* = 198) and in-hospital groups (*n* = 202) were also comparable: Walking difficulty (*n* = 175; HOME: 47.3% vs in-hospital: 46.4%); higher comorbidity (*n* = 188; HOME: 46.1% vs in-hospital: 53.9%); mild cognitive impairment (*n* = 56; HOME: 15.1% vs in-hospital: 13.7%), living alone (*n* = 191; HOME: 47.5% vs in-hospital: 48.0%), received no support from family (*n* = 176; HOME: 54.9% vs in-hospital: 55.8%). No significant differences were found on baseline outcomes measures (NEADL and LLDI) between groups (*p* = .69 to .92), nor for rates of attrition (HOME: 15% vs in-hospital: 16%).

### Variation in response to the HOME intervention

Analyses from linear regression revealed significant interaction effects for intervention by cognitive status for unplanned re-hospitalization (*p* = 0.003) and ED presentations (*p* = 0.021) (see Table 1). Within the mild cognitively impaired subgroup, participants who received the HOME intervention showed lower rates of unplanned hospitalizations at 3 months compared to those who received the in-hospital consultation, and the HOME effect was significant (*p* = 0.027). However, the effect of the HOME intervention on ED presentation did not reach significance within the at-risk subgroup of cognitively impaired participants (*p* = 0.074).

**Table 1:** Interaction effects for Intervention (HOME vs Standard Care) by characteristics of at-risk subgroups at 3 months*

		Interaction			
Dependent Variables	Treatment Effects	Estimate	95%CI**		***p***
Independence in ADLs					
NEADL scale					
*Physical characteristics*					
*Comorbidities* ^*a*^					
*Higher (score 6 to 9)*	0.023	0.304	(−1.468, 2.077)		0.736
*Lower (score < 5)*	− 0.281				
*Ability to walk* ^*b*^					
*With Difficulty (score 5–6)*	−1.115	−1.816	(−3.573, − 0.59)		0.043
*Without Difficulty (score 7)*	0.701				
*Psychological characteristics*					
*Cognitive status* ^*c*^					
*Mildly impaired (3–4 errors)*	0.034	0.287	(−2.353, 2.927)		0.831
*Unimpaired (0 to 2 errors)*	−0.253				
*Social characteristics*					
*Living alone*					
*yes*	−0.879	−1.279	(−3.030, 0.471)		0.152
*no*	0.400				
*Support from family*					
*no*	0.264	0.726	(−1.020, 2.472)		0.414
*yes*	−0.462				
Participation in life roles					
Late life disability Index					
Frequency					
*Physical characteristics*					
*Comorbidities*					
*Higher (score 6 to 9)*	0.359	*1.126*	(−2.643, 4.896)		0.557
*Lower (score < 5)*	−0.767				
*Ability to walk*					
*With Difficulty (score 5–6)*	− 0.837	*−0.925*	(−4.617, 2.766)		0.622
*Without Difficulty (score 7)*	0,088				
*Psychological characteristics*					
*Cognitive status*					
*Mildly impaired (3–4 errors)*	−2.961	*−2.989*	(−8.535, 2.557)		0.290
*Unimpaired (0 to 2 errors)*	0.028				
*Social characteristics*					
*Living alone*					
*yes*	−0.469	−0.228	(−3.888, 3.433)		0.903
*no*	−0.241				
*Support from family*					
*no*	0.698	1.77	(−1.896, 5.437)		0.343
*yes*	−1.072				
Late life disability Index					
Limitation					
*Physical characteristics*					
*Comorbidities*					
*Higher (score 6 to 9)*	−0.742	−0.997	(−6.634, 4.640)		0.728
*Lower (score < 5)*	0.255				
*Ability to walk*					
*With Difficulty (score 5–6)*	−1.348	−3.25	(−8.719, 2.218)		0.243
*Without Difficulty (score 7)*	1.902				
*Psychological characteristics*					
*Cognitive status*					
*Mildly impaired (3–4 errors)*	−4.492	−5.457	(−13.524, 2.610)		0.184
*Unimpaired (0 to 2 errors)*	0.965				
*Social characteristics*					
*Living alone*					
*yes*	−0.465	−0.914	(−6.281, 4.454)		0.738
*no*	0.449				
*Support from family*					
*no*	3.513	6.474	(1.081, 11.866)		**0.019**
*yes*	−2.962				
Number of re-hospitalizations					
Unplanned					
*Physical characteristics*					
*Comorbidities*					
*Higher (score 6 to 9)*	0,074	−0.014	(−0.314, 0.286)		0.928
*Lower (score < 5)*	0.088				
*Ability to walk*					
*With Difficulty (score 5–6)*	0.184	0.238	(−0.073, 0.548)		0.134
*Without Difficulty (score 7)*	−0,054				
*Psychological characteristics*					
*Cognitive status*					
*Mildly impaired (3–4 errors)*	−0.548	−0.692	(−1.138, − 0.245)		**0.003**
*Unimpaired (0 to 2 errors)*	0.144				
*Social characteristics*					
*Living alone*					
*yes*	0.037	−0.036	(−0.334, 0.261)		0.810
*no*	0.073				
*Support from family*					
*no*	0.01	−0.068	(−0.368, 0.231)		0.653
*yes*	0.078				
Number of ED visits					
*Physical characteristics*					
*Comorbidities*					
*Higher (score 6 to 9)*	0.017	−0.129	(−0.452, 0.194)		0.432
*Lower (score < 5)*	0.146				
*Ability to walk*					
*With Difficulty (score 5–6)*	0.128	0.187	(−0.146, 0.521)		0.270
*Without Difficulty (score 7)*	−0.059				
*Psychological characteristics*					
*Cognitive status*					
*Mildly impaired (3–4 errors)*	−0.447	−0.564	(−1.041, − 0.087)		**0.021**
*Unimpaired (0 to 2 errors)*	0.117				
*Social characteristics*					
*Living alone*					
*yes*	−0.011	−0.093	(−0.415, 0.230)		0.572
*no*	0.082				
*Support from family*					
*no*	0.03	0.012	(−0.310, 0.333)		0.942
*yes*	0.018				

*Linear regression models (ANCOVA for independence in ADL and participation in life roles)

**CI: confidence interval

^a^Age-adjusted Charlson Comorbidity Index

^b^Functional Independence Measure score (locomotion item)

^c^Short Portable Mental Status Questionnaire

The HOME effect also differed according to support received from family, as revealed by a significant interaction effect (*p* = 0.019) on participation in life role (i.e. less limitations based on LLDI scores). However, within the at-risk subgroup with no support from family, the effect of the HOME intervention on participation did not reach significance compared to in-hospital consultation (*p* = 0.058).

## Discussion

This study showed that older patients with mild cognitive impairment benefited more from the HOME intervention than those without cognitive impairment unplanned re-hospitalizations at 3 months. Similar findings were obtained by one of the few effectiveness comparative studies measuring the impact of a discharge intervention - Transition Care Model (TCM) - in older adults with cognitive impairment (*n* = 212) [46]. TCM is a nurse-led intervention involving management of medication, pain and nutrition before and after discharge. Consistent with the occupational therapist-led HOME intervention, TCM also used patient-centered and tailored approaches. A significant reduction of re-hospitalization at 3 months (*p* = 0.02) was observed in the group who received TCM (36%), compared to standard care (51%). However, unlike HOME, the benefits of TCM were less important in older adults with cognitive impairment compared to those without [47]. Further information on whether TCM and HOME prevent different root causes of readmissions (e.g. acute vs chronic illness) is needed, which may in turn support combining both interventions and thus provide a novel interdisciplinary approach.

Past qualitative studies on home assessments performed before discharge have suggested that cognitive impairment is an important factor mentioned by therapists (e.g., Atwal et al. [48] and Whitehead et al. [49]). One possible explanation is that assessments in the home enable therapists to observe how the older person interacts with familiar objects – and possibly people - within their own environment. Considering that older adults with mild cognitive impairment tend to perform better in a familiar environment [50], the home assessment makes it possible to distinguish more clearly “real” difficulties (such as not turning off the oven), from those related to a lack of familiarity with the hospital setting (e.g., unknown appliance). Moreover, training in the home environment enables an older person to learn new ways to move around or to use assistive devices in the context in which this knowledge will be applied on a daily basis. It thus requires less cognitive resources compared to when knowledge is needed to be transferred in another context [51], such from the hospital to home. As the familiar environment may trigger old habits, assessments of older persons in their own home may inform about whether they use (or not) spontaneously the strategies taught to them within the hospital environment. Home assessments may also provide unique environmental cues [52], rarely available in the hospital context (e.g., stale foods, untaken pills in the dispenser, damaged appliances, unsafe assistance provided by the caregivers) and, with this additional information, may assist healthcare teams to make appropriate recommendations to better prevent potentially avoidable incidents (e.g., food/drug intoxication, falls, hospital readmissions).

Consistent with the HOME intervention [28], Naylor et al. [46] also found that TCM interventions did not improve independence in ADL for all subgroups of older patients. The lack of significant difference between groups for the NEADL score was still surprising, since unlike TCM, the HOME intervention was developed to improve functional outcomes. Although surprising, this finding is in line with a past review [53] which reported that receiving a home assessment did not demonstrate a benefit on global ADL. Low responsiveness of the NEADL has been reported in some older patients [42], which may support the hypothesis that the outcome measures used in the trial may have lacked sensitivity to change in our sample. Further, it remains plausible that a global measure of ADL may be too blunt when measuring the broad and tailored outcomes required of discharge planning. In the Clemson et al. trial [28], the goals reported by the HOME group were not necessarily focused on ADL performance. As suggested by Liebzeit et al. [54], future studies should explore the relevance to use more personalized outcomes measures, such as goal attainment scales or other methods to understand personal achievement.

The HOME trial also failed to show that the outcomes of walking difficulty and higher comorbidity related to improved outcomes with HOME. Richie et al. [32] previously found that rates of hospitalization may be difficult to reduce in the frailest, and consistent with Whitehead et al. [49], this may suggest that the most dependent patients may not necessarily be the most likely to benefit from an occupational therapy home environment assessment. According to Atwal et al. [55], a home assessment may be perceived as demoralizing, daunting, or may even increase anxiety. Measuring the effects of alternative discharge planning options, such as mobile videoconferencing [56] or virtual home assessments should be considered with these patients, as they may reduce their fatigue while giving access to home environmental cues.

In addition to those mentioned previously for the overall trial [28], this sub-group analysis study has further limitations. First, due to the small sample numbers represented in subgroups, significant interactions may have been underestimated. Second, the sample does not include older adults who required assistance for mobility or those with moderate or severe cognitive impairment, since these potential participants were excluded from the main trial. However, HOME may not be the most appropriate intervention for older adults with dementia due to high rates of institution at discharge (28.8%) compared to those mildly cognitively impaired (7.6%) [57]. Third, outcome measures were solely available at 90 days post-discharge, while larger effects of a similar transition program on hospital and ED readmissions were found after 30 days [58]. It is therefore plausible that we did not capture the full effect of the HOME intervention, even though it is clinically important to assess whether its effects are maintained over time. Finally, in the main trial participant, “at-risk” characteristics were captured individually. Even if our results suggested that older adults with walking difficulty or with high comorbidities do not benefit from HOME, future research still needed to further explore the influence of such patient factors in combination.

## Conclusion

A large proportion of hospitalized older adults are known to experience cognitive impairment [59, 60], common in medical conditions such as chronic obstructive pulmonary disease, heart failure and diabetes [61]. Surprisingly, very few discharge interventions are specifically designed or found to be effective in this subgroup (i.e. older adults with mild cognitive impairments). Our results fill this knowledge gap by suggesting the relevance to apply an occupational therapist-led discharge planning intervention (HOME) to this growing patient population who are at higher risk of poor outcomes [62, 63]. Even if the intervention has not been found effective to improve functional outcomes (ADL), our findings suggest that there may be significant cost savings given the reductions of hospitalization. Cost analyses should be pursued with consideration of how to implement HOME as part of discharge planning for this targeted and growing population [64] and whether some components of the intervention are more “essential” to implement than others. These data may likely be equally as important as the clinical outcomes in changing current acute hospital practices.

## Data Availability

The datasets generated and/or analysed during the current study are available from the corresponding author on reasonable request.

## Abbreviations

ADL: Activities of Daily Living
CCI: Charlson Comorbidity Index
ED: Emergency Department
FIM: Functional Independence Measure
LLDI: Late Life Disability Index
NEADL: Nottingham Extended Activities of Daily Living
RCT: Randomized Control Trial
SPMSQ: Short Portable Mental Status Questionnaire
